# A Typical Bezold Abscess With an Atypical Aetiology

**DOI:** 10.7759/cureus.75397

**Published:** 2024-12-09

**Authors:** Maria Kiakou, Anastasia Dimitriadi, Aikaterini Tsagkovits

**Affiliations:** 1 Ear, Nose, and Throat (ENT), St. George's Hospital, London, GBR; 2 Department of Pathology, General Hospital of Athens "Georgios Gennimatas", Athens, GRC; 3 Department of Otolaryngology, Head and Neck Surgery, General Hospital of Athens "Georgios Gennimatas", Athens, GRC

**Keywords:** middle ear surgery, neck lump, paraganglioma, pathology, skull base surgery

## Abstract

Objective: This study aims to present a case of temporal bone (TBP) paraganglioma with an insidious clinical presentation, deviating significantly from the typical hearing loss and pulsatile tinnitus pattern.

Methods: A 70-year-old lady presented to the emergency department with a five-day history of right progressive later cervical swelling extending to the mastoid region and chronic worsening purulent otorrhea. The clinical and radiological findings confirmed the presence of a chronic middle ear process complicated by a Bezold abscess.

Results: The patient underwent a right-modified radical mastoidectomy with multiple biopsies and drainage of the neck abscess. The histology revealed a temporal bone paraganglioma with the classic Zellballen pattern and characteristic immunohistochemistry. The patient denied any further intervention.

Conclusion: This is the first case of an isolated jugulotympanic paraganglioma presenting as a Bezold abscess. Meticulous intraoperative biopsies and a high grade of suspicion of an underlying pathology are of supreme importance for the diagnosis of these rare entities.

## Introduction

Paragangliomas of the temporal bone (TBP), namely glomus tympanicum and glomus jugulare, are rare tumors with an incidence of approximately 1/1,000,000 per year [1]. They derive from the paraganglionic tissue of the neural crest, presenting in the fifth and sixth decades of life with a sporadic pattern [1]. The head and neck paragangliomas are usually of parasympathetic origin, and they lack endocrine activity in 98% of cases [2]. The main presenting symptoms are conductive hearing loss, pulsatile tinnitus, and rarely lower cranial nerve palsies, while a soft tissue mass in the middle ear is usually the most significant clinical finding [2]. Sometimes they can cause a differential diagnosis dilemma, and they can be confused with a facial nerve schwannoma or a cholesteatoma.

Here, we present the first case of an isolated jugulotympanic paraganglioma presenting as chronic otitis media complicated with a Bezold abscess. The typical symptom of pulsatile tinnitus was completely absent. We aim to highlight the unrivaled importance of tissue sampling in every complicated ear case and the necessity of considering uncommon pathologies even without the presence of typical symptoms.

## Case presentation

History

**Figure 1:**
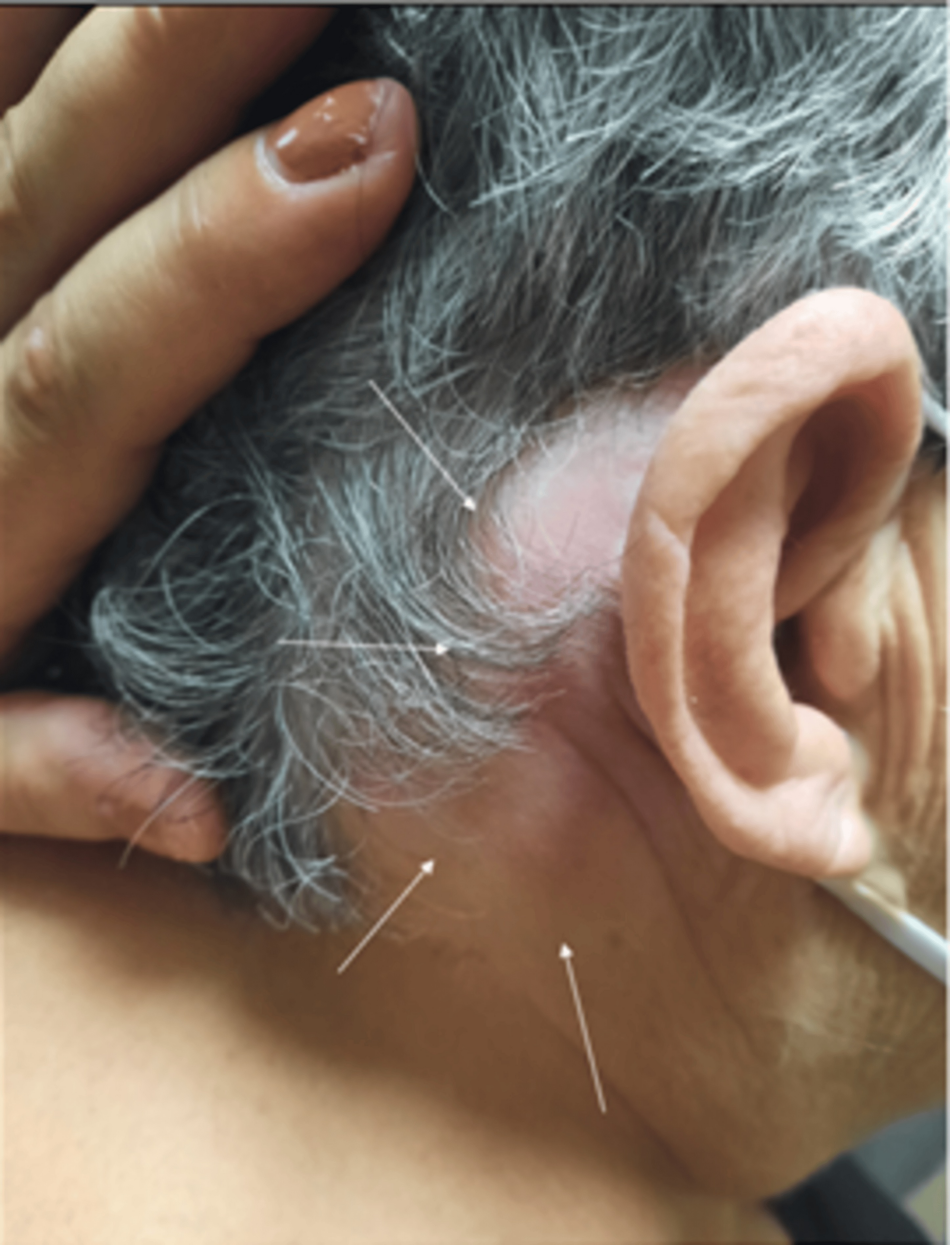
Picture of the right postauricular area of the patient showing a fluctuant swelling, extending from the mastoid to the mid-portion of the sternocleidomastoid muscle. The arrows are pointing the extend of the postauricular erythema and edema.

A 70-year-old, non-smoker woman with a history of well-controlled hypertension presented to the emergency department with a five-day history of right progressive laterocervical swelling extending to the mastoid region and worsening purulent otorrhea (Figure 1). She was otherwise systemically well, apyrexial, and fully alert. She reported hearing loss and intermittent otorrhea for the last two years, without any tinnitus, vertigo, or otalgia.

Examination

The otomicroscopy revealed friable haemorrhagic polypoid tissue completely obstructing the right ear canal and copious purulent discharge. A fairly tender, fluctuant swelling extending from the mastoid to the mid-portion of the sternocleidomastoid muscle was palpable in the ipsilateral neck. The remaining head and neck examination and the brief neurological assessment were normal. The clinical presentation was highly indicative of Bezold’s abscess.

Investigations

**Figure 2:**
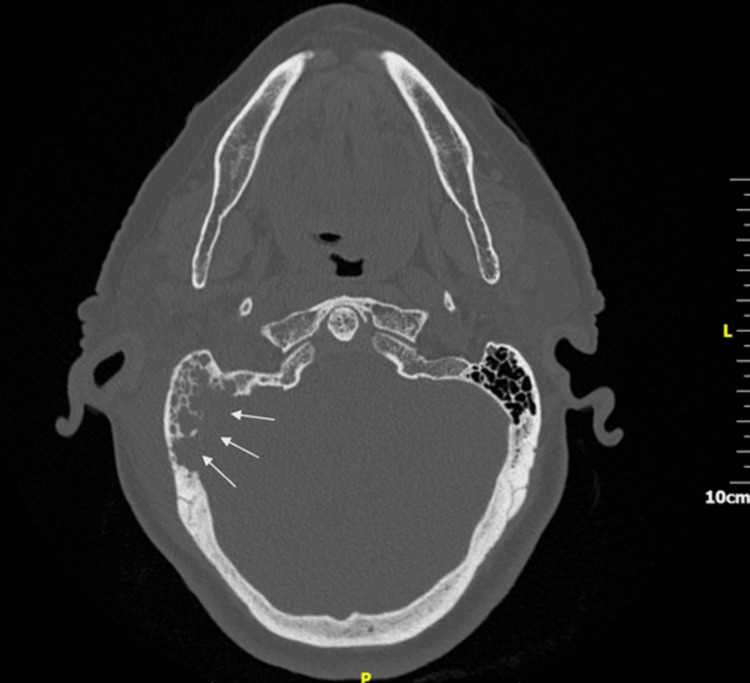
Axial plane of a CT scan temporal bones at the level of the mastoid showing complete opacification of the right middle ear and the mastoid air cells, along with extensive erosion of the mastoid cortical bone, posterior cranial fossa, and sigmoid sinus dural plate (arrows).

**Figure 3:**
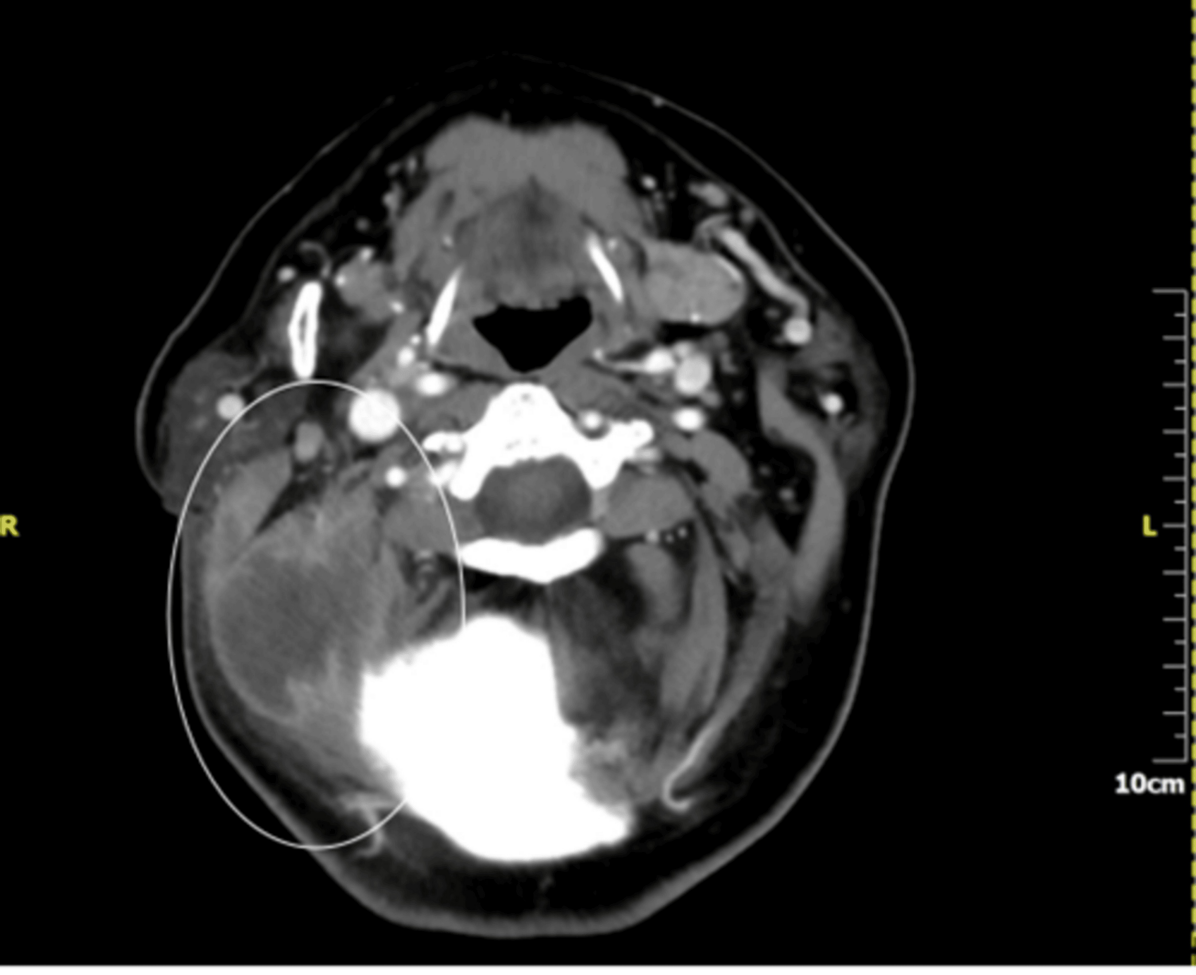
Axial plane of a CT scan of the neck with contrast showing a well-demarcated fluid collection with a peripheral rim enhancement indicating highly an abscess deep to the sternocleidomastoid muscle (highlighted with the white oval shape).

**Figure 4:**
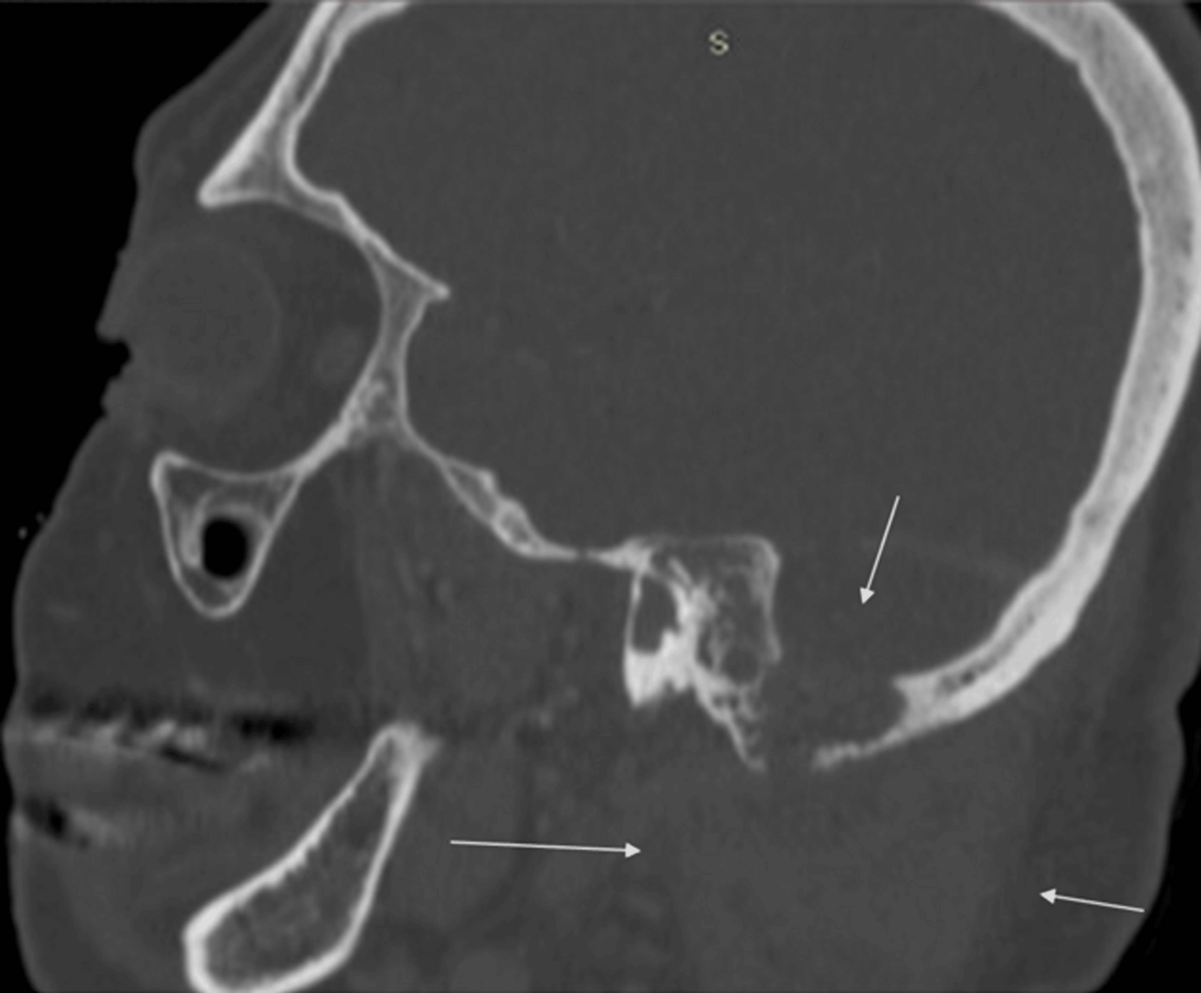
A sagittal plane of the CT scan of the temporal bone showing complete opacification of the right middle ear and the mastoid air cells, along with extensive erosion of the mastoid cortical bone and posterior cranial fossa along with the formation of the neck abscess (contained within the arrows).

A high-resolution CT temporal bone scan and a CT venogram of the head were urgently organised. They showed complete opacification of the right middle ear and the mastoid air cells, along with extensive erosion of the mastoid cortical bone, posterior cranial fossa, and sigmoid sinus dural plate, scutum, and ossicular chain. There was also right sigmoid sinus thrombosis and a lateral neck abscess (Figures 2-4).

The patient underwent a right modified radical mastoidectomy with multiple biopsies and drainage of the neck abscess. Intraoperative findings included a polypoid friable haemorrhagic middle ear mucosa with granulation tissue extending from the mastoid antrum to the hypotympanum and the external auditory canal.

The initial presentation, along with the radiological findings, directed the diagnosis towards a complicated middle ear infection. The patient’s history of longstanding ear discharge was indicative of a chronic rather than acute infection, but the intraoperative presence of excessively bleeding granulomatous middle ear mucosa, with the absence of a pearly cholesteatomatous mass, raised the suspicion of a different pathology.

**Figure 5:**
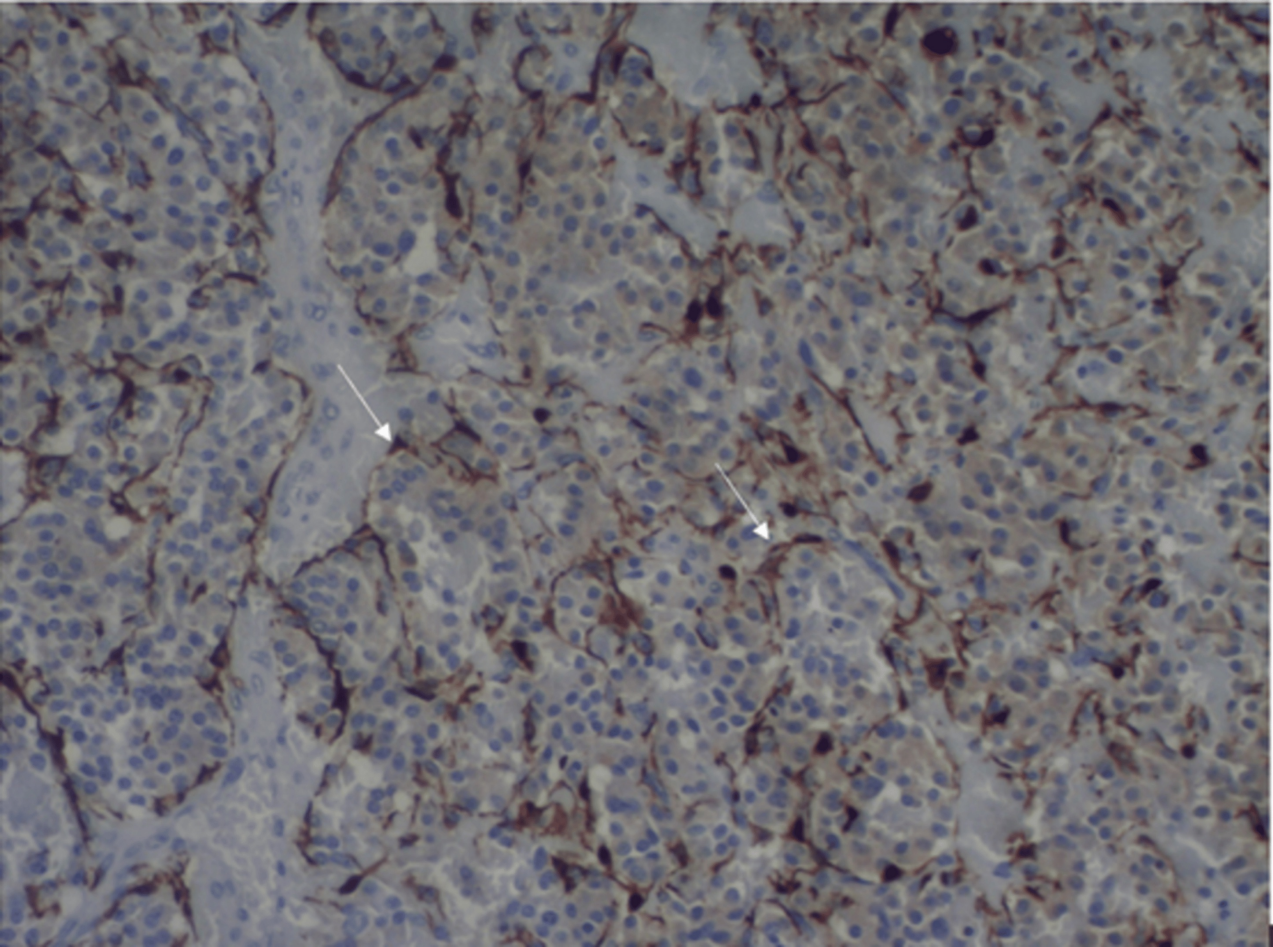
Histological section of the paraganglioma showing a positive S100 immunoreaction of the sustentacular cells (brown staining pointed with white arrows) surrounding the neoplastic nests organised in the classic Zellballen pattern (immunostain S100x100).

The histology revealed a tumour consisting of chief polygonal cells with abundant eosinophilic granular cytoplasm and uniform nuclei, organised in the classic Zellballen pattern (small distinctive nests), separated by prominent fibrovascular stroma. Mild pleomorphism and increased cellularity (>300 nuclei per HPF) were observed, along with rare mitotic activity. The immunohistochemistry markers were negative for CKAE1/AE3 and positive for SYP and CgA in the neoplastic cells. The S-100 marker was positive for the stromal cells and partially positive for the neoplastic cells. The Ki-67 proliferation index was estimated at around 3%. The histological characteristics and the immunohistochemistry markers were typical for a moderately differentiated paraganglioma (Figure 5).

The patient was then considered for further treatment, including surgical excision or radiotherapy, but she denied any further intervention. One year after her initial presentation, she remains asymptomatic with a dry ear.

## Discussion

Head and neck paragangliomas are generally accepted as locally destructive but slowly growing, mostly benign tumors. Middle-aged females are more commonly affected. They originate from parasympathetic fibres located along neurovascular structures, and they do not commonly secrete catecholamines [1]. TBP usually arises along the course of Jacobson’s or Arnold’s nerve, and they are commonly called glomus tympanicum and glomus jugulare, respectively [2]. Even if the term ‘glomus’ is actually inaccurate for the definition of paragangliomas, it is generally accepted and used along with the terms tympanomastoid paraganglioma and jugular paraganglioma [2].

Jugulotympanic paragangliomas usually present with symptomatology related to their local invasive characteristics, with pulsatile tinnitus and hearing loss being the most common ones. However, lower cranial nerves (VII, IX, X, and IX) can also be affected, resulting in symptoms such as facial nerve palsy, dysphagia, hoarseness, and pain [3]. Uncommon presentations with isolated chronic bloody otorrhea or dizziness have also been reported [1,3].

Otomicroscopy may demonstrate a bluish pulsatile mass behind the tympanic membrane, called the ‘rising sun’ sign [3]. They have a metastatic potential, which is determined by their histological appearance and calculated with the Grading of Adrenal Pheochromocytoma and Paraganglioma (GAPP) score (0-10) [1].

Imaging is the cornerstone of the investigation of these tumors. Specifically, a characteristic magnetic resonance imaging (MRI) finding on both T1 and T2-weighted sequences is the “salt and pepper” pattern, which can be quite helpful in guiding the diagnosis [4]. A CT scan is advantageous in evaluating bony erosion of the middle ear surroundings and tumour extension. Glomus tympanicum is characteristically lateral to the promontory, and an intact jugular bulb is necessary for its diagnosis [4]. However, in cases of large tumours invading the jugular bulb and the mastoid cavity, it could be impossible to distinguish a jugular paraganglioma, and the term paraganglioma jugulotympanicum applies [3]. In our case, the tumor was classified as C1, according to the Modified Fisch classification, as it was extending beyond the tympanomastoid cavity, subtly involving the vertical portion of the carotid canal.

In terms of management, small tumors are adequately treated with surgical resection only, while larger paragangliomas (Fisch C and D) usually require adjuvant radiotherapy [5]. The surgical approach highly depends on the tumour extension and the involvement of the jugular bulb. However, in elderly patients, a more conservative approach with partial resection and postoperative radiotherapy is usually considered [5].

It is essential to highlight the complexities faced due to the presentation resembling complicated chronic otitis media. However, cholesteatoma or tumor lesions were also included in the differential diagnosis, given the initial imaging findings. The focus, though, was primarily on addressing the acute phase by draining the neck abscess and managing the primary infection site through a modified radical mastoidectomy. This approach aimed to ensure the timely management of the patient's symptoms while concurrently exploring potential underlying pathologies by obtaining representative biopsies. Post-operatively, the subsequent histological examination revealed the unexpected presence of a moderately differentiated paraganglioma, emphasising the importance of keeping a broad differential and obtaining meticulous biopsies regardless of the presence or absence of typical symptoms.

Moving to further treatment options, the management strategy after the diagnosis was established necessitated careful consideration. While further surgical excision or radiotherapy were viable options for addressing the paraganglioma, the patient opted for conservative management and watchful waiting. This decision underscores the importance of patient preferences and shared decision-making in treatment planning.

## Conclusions

To the best of our knowledge, this is the first reported case of an isolated jugulotympanic paraganglioma presenting as a Bezold abscess. This represents an insidious clinical presentation diverging significantly from the classical symptoms of hearing loss and pulsatile tinnitus. This case report emphasises the importance of keeping a broad differential diagnosis, as typical symptoms do not always indicate a straightforward diagnosis. In our case, the temporal bone paraganglioma was masked by suppuration, even after conducting comprehensive pre-operative assessments and scans, and could have been mistaken for complicated chronic otitis media. However, a quite unexpected diagnosis was revealed after obtaining many peri-operative biopsies.
